# Allosteric regulation of the partitioning of glucose-1-phosphate between glycogen and trehalose biosynthesis in *Mycobacterium tuberculosis*

**DOI:** 10.1016/j.bbagen.2014.09.023

**Published:** 2015-01

**Authors:** Matías D. Asención Diez, Ana M. Demonte, Karl Syson, Diego G. Arias, Andrii Gorelik, Sergio A. Guerrero, Stephen Bornemann, Alberto A. Iglesias

**Affiliations:** a Instituto de Agrobiotecnología del Litoral (UNL-CONICET), Facultad de Bioquímica y Ciencias Biológicas, Paraje El Pozo, S3000ZAA Santa Fe, Argentina; b Department of Biological Chemistry, John Innes Centre, Norwich Research Park, Norwich NR4 7UH, United Kingdom

**Keywords:** ADP-glucose pyrophosphorylase, Glycogen synthase, UDP-glucose pyrophosphorylase, Trehalose-6-phosphate synthase, Phospho*enol*pyruvate, Glucose-6-phosphate

## Abstract

**Background:**

*Mycobacterium tuberculosis* is a pathogenic prokaryote adapted to survive in hostile environments. In this organism and other Gram-positive actinobacteria, the metabolic pathways of glycogen and trehalose are interconnected.

**Results:**

In this work we show the production, purification and characterization of recombinant enzymes involved in the partitioning of glucose-1-phosphate between glycogen and trehalose in *M. tuberculosis* H37Rv, namely: ADP-glucose pyrophosphorylase, glycogen synthase, UDP-glucose pyrophosphorylase and trehalose-6-phosphate synthase. The substrate specificity, kinetic parameters and allosteric regulation of each enzyme were determined. ADP-glucose pyrophosphorylase was highly specific for ADP-glucose while trehalose-6-phosphate synthase used not only ADP-glucose but also UDP-glucose, albeit to a lesser extent. ADP-glucose pyrophosphorylase was allosterically activated primarily by phospho*enol*pyruvate and glucose-6-phosphate, while the activity of trehalose-6-phosphate synthase was increased up to 2-fold by fructose-6-phosphate. None of the other two enzymes tested exhibited allosteric regulation.

**Conclusions:**

Results give information about how the glucose-1-phosphate/ADP-glucose node is controlled after kinetic and regulatory properties of key enzymes for mycobacteria metabolism.

**General significance:**

This work increases our understanding of oligo and polysaccharides metabolism in *M. tuberculosis* and reinforces the importance of the interconnection between glycogen and trehalose biosynthesis in this human pathogen.

## Highlights

• Nucleotide-glucose synthesis in *Mycobacterium tuberculosis* was analyzed.
• The characterization of four enzymes involved in glucose-1P partitioning is reported.
• Mycobacterial ADP-glucose pyrophosphorylase is allosterically regulated.
• Trehalose-6P synthase exhibits higher catalytic efficiency for ADP-glucose.
• Trehalose-6P synthase is activated by fructose-6P.

## 1 Introduction

*Mycobacterium tuberculosis* (*Mtb*) is the causative agent of tuberculosis (TB) in humans, which is one of the most serious pathogenic prokaryotes and one of the leading causes of mortality due to a single infectious agent [1]. *Mtb* is very successful as a pathogen that has adapted itself to survive hostile environments [2]. Many of its metabolic processes have not yet been fully described, and even pathways common to other organisms frequently exhibit distinctive characteristics in *Mtb* [3], [4], which illustrates a metabolic plasticity that helps the organism to adapt and/or survive in the different microenvironments it is challenged with [4], [5], [6], [7], [8], [9]. These particularities in the growth and survival of *Mtb* under nutritionally restrictive conditions (for example in the phagosome) represent attractive targets for new anti-tuberculosis therapies to cope with latent infection of the bacterium [5].

Oligo and polysaccharides are relevant molecules in biology in general, as they are involved in the storage of carbon and energy reserves as well as in establishing cellular structures [10]. Glycogen is a polysaccharide composed of glucose in an α-1,4-linked linear arrangement with α-1,6-branches that serves as a storage molecule in many organisms, including eukaryotes and prokaryotes [11], [12]. Although the particular physiological role of glycogen in bacteria has not been clearly established, it was suggested that its accumulation could give advantages during starvation periods, providing a stored source of energy and carbon surplus [11]. In addition to glycogen, other two polysaccharides in *Mtb* are worth mentioning because of their important physiological roles [13]. One is the extracellular α-glucan, a glycogen-like polymer that is a major component of the capsule that surrounds the bacterial cell and participates in pathogenesis by serving to evade the immune response of the host [14]. The second is methyl glucose lipopolysaccharide (MGLP), an intracellular polymer taking part in modulating the elongation of fatty acids [15], [16], [17]. On the other hand, trehalose (Tre) is also a key carbohydrate in actinobacteria, and its synthesis in mycobacteria was found to be critical because the disaccharide acts as an energy reserve compound and also has structural relevance [18]. Tre is found esterified with different fatty acyl groups in the mycobacterial cell envelope, forming acyltrehaloses [18], [19]. For example, Tre esterified at positions 6 and 6′ by mycolates constitutes the compound known as cord factor, which is a determinant for virulence and survival of *Mtb* in host cells [20]. The synthesis of cord factor has therefore attracted a lot of attention in the development of new anti-TB therapies.

Partitioning of Glc-1P into different metabolic pathways occurs at the point of incorporation of the glycosidic moiety into nucleoside-diphospho-Glc (NDP-Glc) by specific pyrophosphorylases. Subsequently, different glycosyl transferases lead the monosaccharide to the multifaceted routes of carbohydrate anabolism. For the production of storage and structural polysaccharides in bacteria, the synthesis of ADP-Glc and UDP-Glc is most relevant. UDP-Glc is synthesized in a reaction catalyzed by UDP-Glc PPase (EC 2.7.7.9), an enzyme ubiquitously distributed in organisms with a critical role in carbohydrates metabolism [21]. Many important nucleotide sugars such as UDP-xylose, UDP-glucuronic acid and UDP-galactose derive from UDP-Glc [22]. Some of these activated sugars are used to build the glycosidic structure of the bacterial cell wall and capsule or more complex oligo and polysaccharides [22], [23]. UDP-Glc PPases from prokaryotes are not known to be allosterically regulated [24], sharing less than 10% identity with their eukaryotic counterparts [21].

Glycogen synthesis in prokaryotes involves the elongation of an α-1,4-glycosidic chain by glycogen synthase (EC: 2.4.1.21; GSase), using ADP-glucose (ADP-Glc) as the glucosyl donor [11], [12]. In Gram-negative bacteria and cyanobacteria, a key regulatory step in this metabolic route occurs at the level of ADP-Glc synthesis, in the reaction catalyzed by allosteric ADP-Glc pyrophosphorylase (EC: 2.7.7.27; ADP-Glc PPase) [11], [25]. Much less is known concerning what happens in Gram-positive bacteria, with recent reports showing important differences in allosteric regulation [26], [27]. ADP-Glc PPase and GSase are respectively coded by *glgC* and *glgA* which, with the addition of *glgB* (the gene coding for branching enzyme), establish the classical GlgCA pathway for bacterial glycogen synthesis [28].

In *Mtb*, the OtsAB pathway is essential in synthesizing Tre with the use of NDP-Glc by Tre-6P synthase [29], [30], [31]. It has been recently demonstrated [28], [32] that in mycobacteria Tre constitutes a glycogen precursor via a novel pathway (GlgE route), where the dissacharide is converted to maltose and activated to maltose-1P, the latter being transferred to an α-polyglucan molecule. The GlgE pathway thus establishes a metabolic link between Tre and polysaccharides, whose coordinated function and regulation are of relevance for the physiology of the microorganism. GlgE is known to be negatively regulated by phosphorylation [33] and has been genetically validated as a potential drug target [34]. To what extent each of the GlgE and GlgCA pathways contribute to cytosolic glycogen and capsular α-glucan is not yet known.

In general, efforts devoted to the characterization of enzymes related to glycogen metabolism in Gram-positive bacteria are scarce [11]. Recently, our group approached this issue in *Streptomyces coelicolor* [27] and *Streptococcus mutans* [26], where Glc-1P partitioning was understood to be controlled by the allosteric regulation of ADP-Glc PPase. In this work we extend this analysis to the metabolism in *Mtb*, studying the enzymes directing monosaccharides to glycogen and Tre synthesis. We report the molecular cloning and expression of *Mtb* genes coding for ADP-Glc PPase, UDP-Glc PPase, GSase and Tre-6P Sase and characterization of their recombinant products. Kinetic parameters were determined and ADP-Glc PPase regulatory properties were analyzed in detail. Biochemical data are discussed in the context of the metabolism of Tre, glycogen and α-glucan synthesis in mycobacteria, revealing how such a central metabolic node in the production of NDP-Glc is regulated in an important human pathogen.

## 2 Materials and methods

### 2.1 Chemicals

Restriction enzymes were purchased from Promega. All protein standards, antibiotics, isopropyl-β-thiogalactoside (IPTG) and oligonucleotides were obtained from Sigma-Aldrich (Saint Louis, MO, USA). All the other reagents were of the highest quality available.

### 2.2 Bacteria and plasmids

*Escherichia coli* Top 10 F′ cells (Invitrogen) and the pGEM®-T Easy vector (Promega) were used for cloning procedures. Expression of *otsA* was performed in *E. coli* BL21 (DE3) using pRSETA vector (Invitrogen). On the other hand, *glgA*, *glgC* and *galU* genes were expressed in *M. smegmatis* mc^2^155 using the shuttle vector pMIP12 (from Pasteur Institute, Paris, France). Previously, this plasmid was used to obtain a number of proteins from different organisms for immunological purposes [35]. DNA manipulations, *E. coli* and *M. smegmatis* cultures as well as transformations were performed according to standard protocols [36], [37].

### 2.3 Gene amplification

Sequences encoding GSase (*Rv1212c*; *glgA*), ADP-Glc PPase (*Rv1213*; *glgC*), UDP-Glc PPase (*Rv0993*; *galU*) and Tre-6P Sase (*Rv3490*; *otsA*) from *Mtb* H37Rv were amplified by PCR using genomic DNA as the template. Genomic DNA was kindly provided by Drs. Marisa Romano and Fabiana Bigi, from INTA Castelar (Argentina). Primers are listed in Supplemental Table I and were designed for each gene using available genomic information [38], [39] in the GenBank database (http://www.ncbi.nlm.nih.gov/Genbank/index.html). PCR reaction mixtures (50 μl) contained 100 ng of genomic DNA, 2 pg of each primer; 0.2 mM of each dNTP; 2.5 mM Mg^2+^, 5% (v/v) DMSO and 1 U *Pfu* DNA polymerase (Fermentas). Standard conditions of PCR were used for 30 cycles: denaturation at 94 °C for 1 min; annealing at 74 °C for *glgC*, 71 °C for *glgA* and 70 °C for *galU* and *otsA*, for 30 s, and extension at 72 °C for 3 min, with a final extension of 10 min at 72 °C. PCR reaction mixtures were resolved in 1% (w/v) agarose gels and PCR products were purified by means of Wizard SV gel & PCR Clean Up kits (Promega). The amplified genes [previously treated with *Taq* polymerase (Fermentas) and dATP] were cloned into the T-tailed plasmid pGEM-TEasy.

### 2.4 Cloning procedures

Gene identities were confirmed by DNA sequencing (Macrogen, Korea). Afterwards, pGEM-TEasy plasmids harboring *glgC* or *glgA* coding sequences were digested with *KpnI* and *PstI* and the released genes were cloned into pMIP12 to obtain the expression vectors pMIP12/*glgC* and pMIP12/*glgA*. Similarly, pMIP12/*galU* was constructed inserting the gene in the pMIP12 *Bam*HI and *Pst*I sites. Also, pGEM-TEasy/*otsA* plasmid was treated with *Bam*HI and *Hind*III restriction enzymes and subcloned to obtain the pRSET/*otsA* expression vector. In the mycobacterial expression system employed, the recombinant proteins were produced with a C-term His-tag; whereas the Tre-6P Sase was expressed in *E. coli* as a N-terminal His-tagged protein following a strategy similar to that previously reported with slight modifications [40].

### 2.5 Production of ADP-Glc PPase, GSase and UDP-Glc PPase in *M. smegmatis* mc^2^155

Competent *M. smegmatis* mc^2^155 cells were transformed with pMIP12/*glgA*, pMIP12/*glgC* or pMIP12/*galU* according to established protocols [41]. Briefly, competent cells in 200 μl of glycerol 10% (v/v) were mixed with 200 ng of plasmidic DNA in a 2 mm cuvette (HYBAID). Electroporation was performed in a Thermo CelljecT Duo (HYBAID; set at R = 335Ω, V = 2.5 kV and Φ = 15 μF). Cells were harvested in 1 ml of LB-0.05 % Tween 80 and incubated for 3 h at 37 °C without shaking. Positive transformants were selected by plating *M. smegmatis* on LB-Tween-agar containing 50 μg/ml of kanamycin. Expression in *M. smegmatis* was performed in LB-low salt (5 g/l of NaCl) medium supplemented with 0.05% Tween 80 and 50 μg/ml of kanamycin. First, a 10 ml “starter culture” was grown for 24 h and used to inoculate 1 l of the same medium. Expression cultures were incubated in an orbital shaker at 200 rpm and grown for 96 h at 37 °C. Cells were harvested by centrifugation at 5000 × *g* for 10 min and stored at − 20 °C until processing.

### 2.6 Production of Tre-6P Sase in *E. coli*

Competent *E. coli* BL21 (DE3) cells were transformed with pRSET/*otsA* plasmid. Protein production was carried out using 2 l of LB supplemented with 100 μg/ml ampicillin. Cells were grown at 37 °C and 250 rpm until OD_600_ reached ~ 0.6 and induced for 16 h at 20 °C with 0.2 mM IPTG. Cells were harvested by centrifugation at 5000 × *g* for 10 min and stored at − 20 °C until use.

### 2.7 Purification of recombinant proteins

Purification procedures were carried out at 4 °C. Cells for each expressing culture were harvested by centrifugation at 5000 ×*g* for 10 min, resuspended in *Buffer A* [20 mM Tris–HCl, pH 8.0, 400 mM NaCl and 10 mM imidazole] and disrupted by sonication on ice (5 pulses of 30 s with 60 s intervals). The suspension was centrifuged twice at 10,000 ×*g* for 10 min and the supernatant (crude extract) was loaded on a 1 ml HisTrap column (GE Healthcare) previously equilibrated with *Buffer A*. The recombinant protein was eluted with a linear gradient from 10 to 300 mM imidazole in *Buffer A* (50 volumes), and fractions containing the highest activity were pooled and concentrated to 2 ml. Active ADP-Glc PPase and UDP-Glc PPase fractions were dialyzed against *Buffer B* [50 mM MOPS pH, 8.0, 0.1 mM EDTA, 5 mM MgCl_2_, 5% (w/v) sucrose and 10% (v/v) glycerol]. GSase was dialyzed against buffer containing triethanolamine-HCl 20 mM, pH 8.0, and 20% (v/v) glycerol and Tre-6P Sase was dialyzed against a buffer containing 20 mM Tris–HCl, pH 8.0, and 10% (v/v) glycerol. In these conditions the enzymes were stored at − 80 °C until use, remaining fully actives for at least 3 months.

### 2.8 Protein methods

Protein concentration was determined by the modified Bradford assay [42] using BSA as a standard. Recombinant proteins and purification fractions were defined by sodium dodecyl sulfate polyacrylamide gel electrophoresis (SDS-PAGE) according to [43]. Gels were loaded with 5 to 50 μg of protein per well and stained with Coomassie-Brilliant Blue. Western blotting was performed using standard techniques [37]. Proteins in the gel were blotted onto PVDF membranes using a Mini-PROTEAN II (Bio-Rad) apparatus. The membrane was blocked 2 h at room temperature and subsequently incubated overnight with primary antibody at 4 °C. Then, membranes were incubated with rabbit anti-IgG conjugated to peroxidase (Sigma) during 1 h at 25 °C. Detection was carried out with 3,3-diaminobenzidine and hydrogen peroxide (Sigma) in 50 mM Tris–HCl, pH 8.0, and 150 mM NaCl.

Antibodies raised against *Mtb* ADP-Glc PPase or *Xhantomonas campestris* UDP-Glc PPase [44] were produced in our lab according to established methods [45] and used as primary antibodies. They were purified from rabbit sera by consecutive precipitation steps with ammonium sulfate 50% and 33% (twice) saturated solutions. After that, antibodies were resuspended in TBS buffer (Tris–HCl pH 8.0, and NaCl 150 mM) and desalted using an ultrafiltration device with a 30 kDa cut-off (Amicom).

### 2.9 Enzyme activity assays

ADP-Glc PPase and UDP-Glc PPase activities were determined at 37 °C in both NDP-Glc pyrophosphorolysis (assay A) and synthesis (assay B) directions.

Assay A. Pyrophosphorolysis of ADP-Glc or UDP-Glc was followed by the formation of [^32^P]ATP or [^32^P]UTP, respectively, from [^32^P]PP_i_, as previously described [46]. Reaction mixtures contained 50 mM MOPS buffer, pH 8.0, 5 mM MgCl_2,_ either 2 mM ADP-Glc or 1 mM UDP-Glc (depending of the enzyme analyzed), 1 mM [^32^P]PP_i_ (3000 cpm/nmol), 10 mM NaF, 0.2 mg/ml BSA and enzyme in a final volume of 150 μl. Reactions were started with ^32^PP_i_ addition and after 10 min of incubation at 37 °C were stopped with 1 ml of cold 10% (v/v) trichloroacetic acid.

Assay B. Synthesis of ADP-Glc or UDP-Glc was assayed by following the formation of P_i_ (after hydrolysis of PP_i_ by inorganic pyrophosphatase) with the highly sensitive colorimetric method previously described [47]. The reaction mixture contained 50 mM MOPS, pH 8.0, 5 mM MgCl_2_, either 2 mM ATP or 1 mM UTP (depending of the enzyme analyzed), 0.2 mg/ml BSA, 0.0005 U/μl yeast inorganic pyrophosphatase and appropriately diluted enzyme. Assays were initiated by addition of Glc-1P in a total volume of 50 μl. The reaction mixture was incubated for 10 min at 37 °C and terminated by adding the Malachite Green reagent. The complex formed with the released P_i_ was measured at 630 nm in a Multiskan Ascent microplate reader (Thermo Electron Corporation). The conversion of substrates to the expected products was confirmed using proton NMR spectroscopy.

Alternatively, assay B was replaced by the radiometric coupled assay method [48], measuring the synthesis of ADP-[^14^C]Glc from [^14^C]Glc-1P and ATP. The standard reaction mixture contained 100 mM MOPS buffer, pH 8.0, 10 mM MgCl_2_, 1 mM [^14^C]Glc-1P (100–1000 cpm/nmol), 1.5 mM ATP, 0.5 U/ml inorganic pyrophosphatase, 0.2 mg/ml BSA and enzyme in a total volume of 0.2 ml. Reaction mixtures were incubated for 10 min at 37 °C and terminated by heating in a boiling-water bath for 1 min. The ADP-[^14^C]Glc was then converted to [^14^C]glycogen by the addition of *E. coli* GSase and non-radioactive glycogen as a primer. Glycogen formed was precipitated and washed, and the radioactivity measured in a scintillation counter.

Tre-6P Sase. Synthesis of Tre-6P from NDP-Glc and Glc-6P was assayed by measuring NADH formation at 340 nm via the coupled spectrophotometric method previously utilized for other glycosyl transferases [40], [49], [50]. The standard media contained 50 mM MOPS, pH 8.0, 5 mM MgCl_2_, 5 mM MnCl_2_, 0.3 mM phospho*enol*pyruvate, 0.3 mM NADH, 2.5 mM NDP-Glc, 5 mM Glc-6P, 2 U pyruvate kinase, 2 U lactate dehydrogenase and 0.2 mg/ml BSA and appropriately diluted enzyme in a final volume of 100 μl. Reactions were incubated at 37 °C in a 96-well microplate and oxidation of NADH was followed at 340 nm using a Multiskan Ascent microplate reader (Thermo Electron Corporation). The conversion of substrates to the expected products was confirmed using proton NMR spectroscopy.

GSase. The assay was conducted as described in [51], using a solution that contained 1 mM ADP-[^14^C]Glc (500–1500 cpm/nmol), 10 mM MgCl_2_, 2.5 mg/ml rabbit liver glycogen, 50 mM bicine–NaOH, pH 8.0, and 0.2 mg/ml BSA in a total volume of 100 μl. Assays were started by adding 20 μl of GSase dissolved in 20 mM triethanolamine-HCl, pH 8.0. GSase activity was alternatively measured with the same procedure used for Tre-6P Sase, but replacing Glc-6P by 2.5 mg/ml rabbit liver glycogen, according to [50]. The conversion of substrates to the expected products was confirmed using proton NMR spectroscopy.

One unit of activity (U) is defined as the amount of enzyme catalyzing the formation of 1 μmol of product per min under the conditions described.

### 2.10 Calculation of kinetic constants

Saturation curves were defined by assaying enzyme activity at different concentrations of the variable substrate or effector with saturating levels of the other components. The experimental data were plotted as enzyme activity (U/mg) *versus* substrate (or effector) concentration (mM), and kinetic constants were determined by fitting the data to the Hill equation as described elsewhere [52]. Fitting was performed with the Levenberg–Marquardt nonlinear least-squares algorithm provided by the computer program Origin™. Hill plots were used to calculate the Hill coefficient (*n*_H_), the maximal velocity (*V*_max_), and the kinetic constants that correspond to the activator or substrate concentrations giving 50% of the maximal activation (*A*_0.5_), or velocity (*S*_0.5_). All kinetic constants are the mean of at least three independent sets of data, which were reproducible within ± 10%.

## 3 Results

### 3.1 Molecular cloning of genes from *M. tuberculosis* H37Rv and production of soluble recombinant proteins

To gain knowledge on the properties of key enzymes involved in carbohydrates metabolism in mycobacteria, we designed experimental strategies to recombinantly produce the proteins with high purity. Using the information available from the genome project of *Mtb* (strain H37Rv) [38], we amplified four genes defining the metabolic node involving Glc-1P and NDP-Glc from genomic DNA. The genes thus cloned were *glgC* (1215 bp), *glgA* (1164 bp), *galU* (912 bp) and *otsA* (1503 pb), respectively encoding ADP-Glc PPase, GSase, UDP-Glc PPase and Tre-6P Sase. The *otsA* gene could be expressed in *E. coli* BL21 (DE3) using the pRSETB/*otsA* construct to produce the mycobacterial Tre-6P Sase in a soluble and active form, following the strategy previously reported for this enzyme [40]. The expression was conducted using 0.1 mM IPTG during 4 h at 23 °C, conditions under which most of the protein was in the insoluble fraction, but reaching a level of soluble and active enzyme sufficient for its purification.

It has been reported that many mycobacterial proteins are particularly recalcitrant to heterologous expression in *E. coli* cells as soluble forms [53], [54]. This was our experience with the production of the other three proteins (besides Tre-6P Sase) characterized in this study. The genes *glgA*, *glgC* and *galU* could not be expressed in *E. coli* to give soluble proteins using pRSETB or pET vectors, even when different expression conditions were attempted, as detailed by Supplemental Fig. 1 for production of ADP-Glc PPase. A high level of production of the recombinant proteins was observed but only in the insoluble fraction, even when temperature and time of expression were modified. Similar results were obtained with different growth media or using a strategy of co-expression with chaperones; conditions that usually are effective to overcome the expression of insoluble proteins from actinobacteria [27]. All expression conditions tested were unsuccessful in obtaining significant levels of ADP-Glc PPase in a soluble form that could be detected using the activity assay or with specific antibodies in western blots. Despite being able to produce a little soluble protein through denaturation and refolded according to established protocols [55], [56], [57], it was not possible to obtain any detectable activity.

**Fig. 1:**
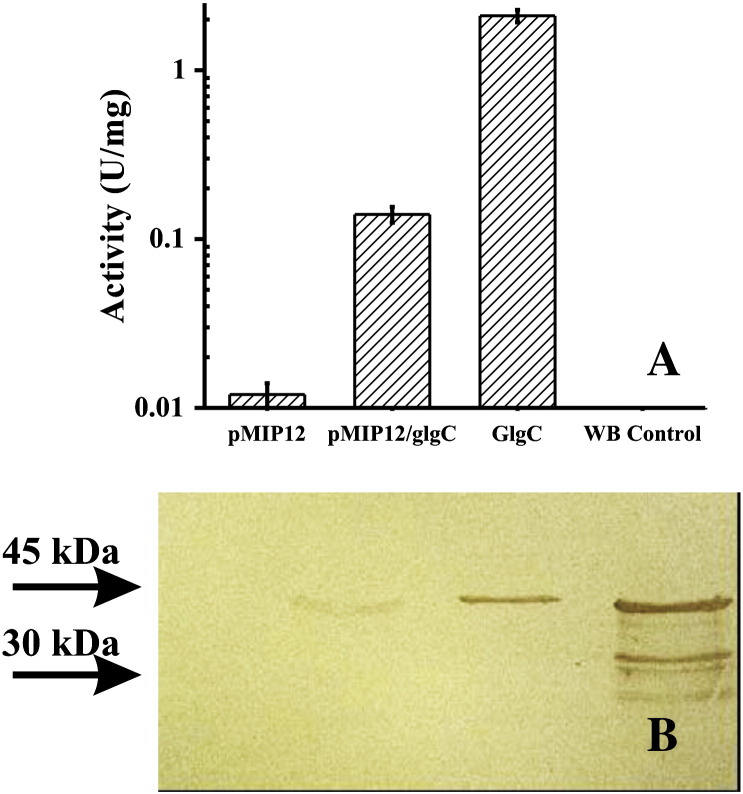
Expression of *Mtb* H37Rv ADP-Glc PPase with the vector pMIP12 in *M. smegmatis* mc^2^155. (A) Activity histogram of soluble samples. (B) Immunodetection of ADP-Glc PPase of corresponding samples in A after SDS-PAGE and immunobloting. The lanes are defined as follows: pMIP12, crude extracts from *M. smegmatis* mc^2^155 cells transformed with pMIP12 (empty vector control); pMIP12/glgC, crude extracts from *M. smegmatis* mc^2^155 cells transformed with pMIP12/*MtbglgC*; GlgC, purified ADP-Glc PPase; WB control, denatured/solubilized pellet from *E. coli* BL21cells transformed with pET19/*MtglgC*. Samples were assayed for activity in the direction of ADP-Glc synthesis, as stated under Materials and methods for Assay B.

To overcome problems associated with expression of mycobacterial proteins in *E. coli*, we selected the alternative of using a more related bacterium as a surrogate and a more compatible host. Thus, *glgC*, *glgA* and *galU* genes from *Mtb* H37Rv were cloned into pMIP12 for expression in *M. smegmatis* mc^2^155 cells. Using this procedure, recombinant ADP-Glc PPase was produced as a soluble protein (Fig. 1) that could be detected in western-blots and further purified. Also, the specific activity of the enzyme in crude extracts was about one order of magnitude higher than that detected in cells of *M. smegmatis* mc^2^155 transformed with pMIP12 alone (empty vector control). This strategy was also successful for the expression of UDP-Glc PPase and GSase.

Either using the pMIP12 or the pRSETB vector system, the four enzymes from *Mtb* were produced as proteins having a fused His-tag respectively at the C-terminus (ADP-Glc PPase, UDP-Glc PPase and GSase) or the N-terminus (Tre-6P Sase). The use of the His-tag was convenient in two ways. First, it enabled the separation of each recombinant enzyme from its respective ortholog protein occurring in the host cell. Secondly, it allowed the purification in one-step by IMAC to give each enzyme with a high degree of purity as determined by SDS-PAGE (Supplemental Fig. 2). The values of specific activity of the purified enzymes were 3.3 and 2.7 U/mg for ADP-Glc PPase and UDP-Glc PPase, respectively (both determined in the direction of NDP-Glc synthesis using Assay B), 0.2 U/mg for GSase and 1.6 U/mg for Tre-6P Sase (in the direction of Tre-6P synthesis).

### 3.2 Kinetic properties of the recombinant mycobacterial enzymes

Table 1 summarizes the kinetic parameters determined for recombinant ADP-Glc PPase, UDP-Glc PPase and GSase. The pyrophosphorylases were characterized in both directions of catalysis (NDP-Glc synthesis and pyrophosphorolysis) and using Mg^2+^ as an essential cofactor. *Mtb* ADP-Glc PPase exhibited between 2- and 3-fold lower affinity for ATP and Glc-1P compared with those reported for the enzyme from the related actinobacteria *S. coelicolor* [27] and *M. smegmatis* [58]. However, it had a *V*_max_ of ~ 3 U/mg, which is almost 20-fold higher than that reported for the *S. coelicolor* enzyme [27]. The *Mtb* ADP-Glc PPase gave saturation kinetics for Mg^2+^, ATP, ADP-Glc and Glc-1P with different degrees of sigmoidicity. The behavior of PP_i_ was unusual, since no saturation could be achieved for the substrate in the concentration range evaluated (up to 2 mM PP_i_, where higher concentrations can precipitate in the assay medium) and thus the affinity parameter could only be estimated (Table 1).

**Table 1:** Kinetic parameters for ADP-Glc PPase, UDP-Glc PPase and GSase from *Mtb*. Values represent means of three independent experiments.

Enzyme	Substrate	*S*_0.5_ (mM)	*n*_H_	*V*_max_ (U/mg)
*ADP-Glc PPase*				
*Assay A*	ATP	1.20 ± 0.08	2.2	3.32 ± 0.11
	Glc-1P	1.07 ± 0.09	1.4	
	Mg^2+^	1.29 ± 0.13	2.3	
*Assay B*	ADP-Glc	0.76 ± 0.09	2.1	1.41 ± 0.08
	PP_i_	> 2	–	
	Mg^2+^	0.81 ± 0.09	3.0	

*UDP-Glc PPase*				
*Assay A*	UTP	0.10 ± 0.02	1.2	2.52 ± 0.09
	Glc-1P	0.13 ± 0.01	1.5	
	Mg^2+^	0.46 ± 0.07	2.9	
*Assay B*	UDP-Glc	0.76 ± 0.04	1.0	1.63 ± 0.11
	PP_i_	0.61 ± 0.05	1.6	
	Mg^2+^	0.47 ± 0.06	3.6	

*GSase*				
	ADP-Glc	3.95 ± 0.12	2.5	0.21 ± 0.02
	Glycogen	0.30 ± 0.02 (mg/ml)	2.1	

In the two directions of catalysis (UDP-Glc synthesis and pyrophosphorolysis), the UDP-Glc PPase from *Mtb* showed similar specific activities (~ 2.5 and ~ 1.6 U/mg, respectively), with affinities for the substrates and the cofactor between 0.1–0.8 mM (Table 1). Interestingly, results indicate that the *Mtb* enzyme reported herein is 25-fold more active for UDP-Glc synthesis than the same enzyme characterized after its recombinant expression using *E. coli* as a host [59], consistent with expression in the Gram-negative host being problematic. In the this direction of catalysis, the *V*_max_ determined for *Mtb* UDP-Glc PPase is two orders of magnitude lower that that reported for the homologous enzyme from *S. coelicolor* [27]. However, the affinity for its substrates is ~ 10-fold higher than those of ADP-Glc PPase from *Mtb* and of UDP-Glc PPase in crude extracts of *M. smegmatis* [58] (Table 1). Concerning the *Mtb* GSase, its kinetic parameters shown in Table 1 are similar to those reported for the enzyme from the related Gram-positive bacterium *S. coelicolor* [27], except for a 30-fold lower affinity for ADP-Glc exhibited by the former.

Both pyrophosphorylases and the GSase from *Mtb* were highly specific for the nucleotide substrates. Thus, GSase used ADP-Glc to elongate glycogen and no activity was detected with UDP-Glc up to 10 mM. Furthermore, UDP-Glc was not an inhibitor of the reaction with ADP-Glc. The analysis of different NTPs (ATP, UTP, GTP, dTTP) as substrates of ADP-Glc PPase and UDP-Glc PPase (assayed up to 5 mM) showed complete specificity for the use of ATP and UTP, respectively. These results are consistent with the functional operation of the pathways for glycogen biosynthesis (via ADP-Glc, the GlgCA route) or the metabolism of structural oligo and polysaccharides as well as sugar inter-conversion (by using UDP-Glc) in bacteria [11], [12].

**Fig. 2:**
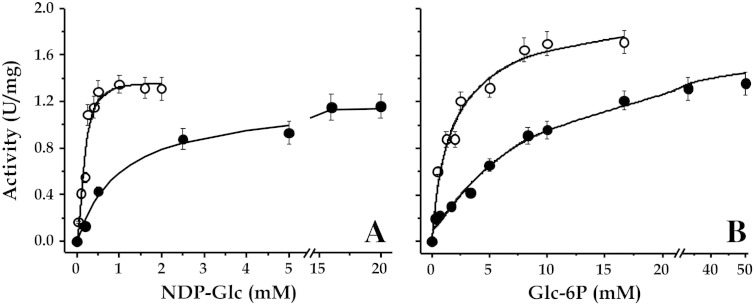
Saturation plots of *Mtb* Tre-6P Sase with the substrates NDP-Glc (A) and Glc-6P (B). The effect of ADP-Glc (empty circles) and UDP-Glc (filled circles) concentrations were assessed in the presence of 1.5 mM Glc-6P and that of Glc-6P in the presence of 1 mM ADP-Glc or 5 mM UDP-Glc.

The recombinant *Mtb* Tre-6P Sase was found to use both, UDP-Glc and ADP-Glc as substrates for synthesis of Tre-6P (Fig. 2). Although the enzyme reached similar *V*_max_ values with UDP-Glc and ADP-Glc (52% higher with the latter), major differences arise when *S*_0.5_ values are compared (Table 2 and Fig. 2A). Thus, the affinity of the enzyme toward ADP-Glc was found to be one order of magnitude higher than for UDP-Glc. In addition, when the activity was assayed in the presence of saturated concentration of ADP-Glc, Tre-6P Sase exhibited 4.3-fold lower *S*_0.5_ for Glc-6P compared with activity using UDP-Glc (Table 2 and Fig. 2B). Saturation plots for Glc-6P with either NDP-Glc were hyperbolic, while both ADP-Glc and UDP-Glc gave slight positive cooperativity. We also tested Tre-6P activity with Fru-6P, Fru-1,6-bisP, mannose-6P or sorbitol-6P instead of Glc-6P in the presence of APG-Glc or UDP-Glc. The mycobacterial enzyme was strictly specific for Glc-6P in accordance with previous work [40], [60]. Nevertheless, this is the first kinetic analysis of the *Mtb* Tre-6P Sase showing the preferential use of ADP-Glc as the main donor substrate.

**Table 2:** Kinetic parameters for Tre-6P Sase from *Mtb*.

Substrate	*S*_0.5_ (mM)	*n*_H_	*V*_max_ (U/mg)
ADP-Glc	0.14 ± 0.01	1.3	1.37 ± 0.11
Glc-6P	1.43 ± 0.08	1.0	1.84 ± 0.08
UDP-Glc	1.54 ± 0.07	1.4	1.19 ± 0.10
Glc-6P	6.21 ± 0.12	0.9	1.28 ± 0.09

### 3.3 Regulatory properties of the recombinant mycobacterial enzymes

ADP-Glc PPases from different sources are allosterically regulated by key metabolites belonging to the principal carbon assimilation route in the respective organism [11], [25]. It has been established in many bacteria that regulation of the enzyme is critical to determine the track of Glc-1P in cellular carbon metabolism [11]. To assess the issue in *Mtb,* we explored the potential regulatory properties of the recombinant enzymes in this study. Activation-inhibition assays were performed for the *Mtb* ADP-Glc PPase with compounds that are known to be important effectors of the enzyme in various organisms [11], [25], [27]: pyruvate, phospho*enol*pyruvate (PEP), 3-phosphoglycerate, Fru-6P, Fru-1,6-bisP, ribose-5P, Glc-6P, mannose-1P, mannose-6P, AMP, ADP, Pi, NAD(P)^+^, and NAD(P)H. The concentration of the effectors analyzed ranged between 0.05 and 5 mM while substrates were maintained at saturating concentrations. PEP and Glc-6P were activators of *Mtb* ADP-Glc PPase giving up to a 3-fold increase in activity. Also, a very weak activation was observed with Fru-6P (1.3-fold at 5 mM). On the other hand, AMP and ADP showed slight inhibitory effects, diminishing the activity up to 50%; while P_i_, a common inhibitor for ADP-Glc PPases (e.g. the enzyme from *S. coelicolor*), had no effect on the *Mtb* enzyme. It is worth mentioning that both PEP and Glc-6P were reported as activators of the ADP-Glc PPase from *M. smegmatis* [58] and *S. coelicolor* [27], thus suggesting a common activation in these phylogenetically related actinobacteria. Conversely, *Mtb* UDP-Glc PPase and GSase were insensitive to the metabolites tested, in agreement with the lack of regulatory properties already reported for both enzymes from prokaryotes [21], [23], [24], [27], [44], [61], including that from *M. smegmatis* [58].

**Fig. 3:**
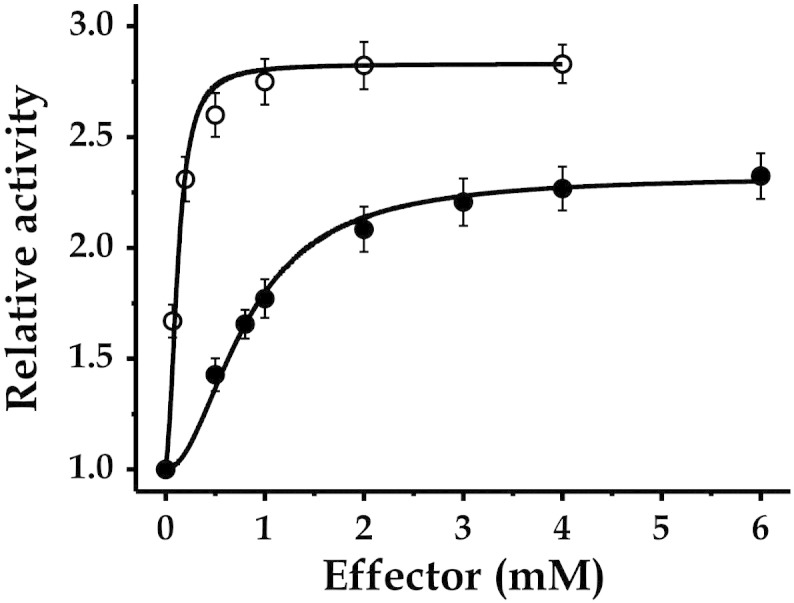
Saturation plots for the allosteric activation of ADP-Glc PPase from *Mtb*. Curves were made in the ADP-Glc synthesis direction of catalysis with Assay A in the presence of 2 mM PEP (empty circles) or 2 mM Glc-6P (filled circles).

Characterization of the response of *Mtb* ADP-Glc PPase to effectors is particularly important for a deeper comprehension of carbon partitioning at the metabolic Glc-1P node as well as a better understanding of glycogen synthesis by the mycobacterial classical GlgCA pathway. Saturation kinetics for Glc-6P and PEP indicated that the effectors enhanced the activity of the *Mtb* enzyme by 2.4- and 2.9-fold, with *A*_0.5_ values of 0.87 and 0.09 mM, respectively (Fig. 3). However, the effect of both compounds was not only exerted on the enzyme *V*_max_, but they also decreased values of *S*_0.5_ for substrates. Results in Table 3 highlight the importance of Glc-6P in the activation of the mycobacterial ADP-Glc PPase, since the effector increased by ~ 5-fold the enzyme's apparent affinity for Glc-1P and ATP, with a consequent enhancement of the *k*_cat_/*S*_0.5_ ratio (equivalent to *k*_cat_/*K*_m_ or catalytic efficiency for hyperbolic kinetics) by one order of magnitude. The effect of PEP mainly enhanced the enzyme's affinity for ATP and increased by ~ 7-fold the catalytic efficiency with this substrate (Table 3). These results suggest that the reaction catalyzed by ADP-Glc PPase would be under allosteric regulation by key metabolites of the carbon metabolism in *Mtb*, which is a characteristic common to other bacteria [11], [12], [25]. Concerning the regulatory properties of the mycobacterial enzyme, they resemble those reported for *S. coelicolor* ADP-Glc PPase, which also has Glc-6P and PEP as its main activators [27].

**Table 3:** Analysis of the activation of ADP-Glc PPase from *Mtb*.

Condition	Substrate	
	ATP	Glc-1P
*Relative affinity*^a^		
No effector	1.0	1.0
2 mM Glc-6P	5.2	4.6
2 mM PEP	2.3	1.2

*Relative activity* b		
No effector	1.0	1.0
2 mM Glc-6P	2.5	2.4
2 mM PEP	2.9	3.0

*Relative catalytic efficiency*^c^		
No effector	1.0	1.0
2 mM Glc-6P	13	11
2 mM PEP	6.7	3.6

a Calculated as the ratio of *S*_0.5_ values obtained in absence over in presence of the effector.

b Calculated as the ratio of *V*_max_ values obtained in presence over in absence of the effector.

c Calculated as the ratio between (*k*_cat_/*S*_0.5_) values obtained in presence over in absence of the effector.

**Fig. 4:**
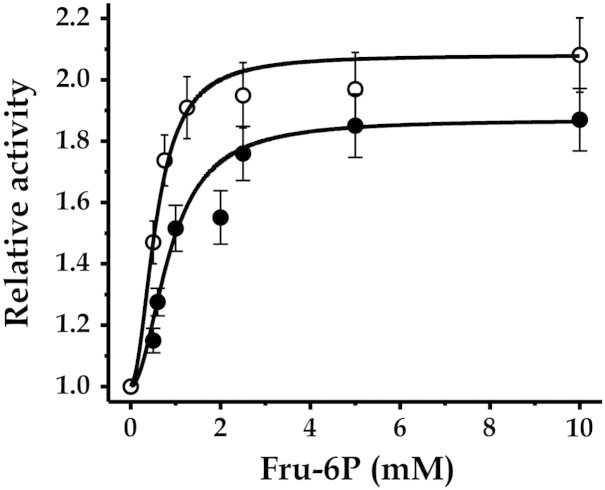
Saturation plots for the activation of *Mtb* Tre-6P Sase by Fru-6P. Curves were obtained with 10 mM Glc-6P and either 2 mM ADP-Glc (empty circles) or 10 mM UDP-Glc (filled circles).

The recombinant *Mtb* Tre-6P Sase was also analyzed for allosteric regulation by metabolites. Only Fru-6P had an effect, activating the enzyme by 2-fold when assayed either with ADP-Glc (2 mM) or UDP-Glc (10 mM). Saturation kinetics shown in Fig. 4 indicate that the enzyme exhibited a higher apparent affinity for the activator when the substrate was ADP-Glc compared with UDP-Glc, with *A*_0.5_ values for Fru-6P determined to be 0.33 and 1.1 mM, respectively. The activating effect was mainly on *V*_max_, as the values of *S*_0.5_ for the enzyme substrates were not significantly modified when determined in the presence of Fru-6P (data not shown). Previous reports on the regulation of mycobacterial Tre-6P Sase only referred the activation by polyanions acting at relatively high concentrations [60], [62]. This effect was analyzed in detail for the *Mtb* enzyme by Pan and collegues [40], describing that both the recombinant Tre-6P Sase and the one purified from crude extracts have similar properties in the presence or absence of polyanions. In addition, recent work characterizing both Tre-6P Sase isoforms from *R.opacus* showed that one of them is strongly dependent on heparin activation (OtsA2), but not the other (OtsA1) [63]. We have focused on regulation by metabolic intermediates and, although modest, the activation of the enzyme by the glycolytic intermediate Fru-6P as described in the present study could have a physiological role in mycobacteria.

## 4 Discussion

Regulation of glycogen synthesis has been extensively analyzed in Gram-negative bacteria (mainly in *E. coli*) [11], [12], but markedly scarce information is available concerning other prokaryotes. Recently, the occurrence of the polysaccharide in mycobacteria and actinobacteria acquired additional interest, because a novel GlgE pathway relating Tre and glycogen metabolism was discovered with implications in the development of new drugs against TB [28], [32], [33], [64]. It was recently demonstrated that in *Mtb* the maltosyltransferase GlgE is negatively regulated by phosphorylation of Ser/Thr residues [33]. However, the kinetic and regulatory characterization of the NDP-Glc-related enzymes in the classical GlgCA pathway for glycogen synthesis and for Tre biosynthesis in *Mtb* have not been performed. This is critical for an understanding of the relationships between pathways leading to the synthesis of oligo and polysaccharides that serve as structural components, carbon reserves and bioactive compounds in the pathogen. All of the Glc polymer pathways relevant to this work form a complex network [28]. For example, the GlgE and GlgCA pathways have potentially common intermediates (e.g. maltooligosaccharides) and Tre can be regenerated from glycogen via the TreXYZ pathway. Nevertheless, all of these polysaccharides and Tre must first be synthesized via either ADP-Glc or UDP-Glc from Glc-1P.

In the present work we report the molecular cloning, recombinant expression and characterization of four enzymes that define the partition of Glc-1P into different anabolic routes of carbohydrates in *Mtb*. Thus, recombinant Tre-6P Sase could be produced by expression of the gene in *E. coli*, which was of utility to define properties of the enzyme that were not identified in previous studies [40], [60] and that are of value for a better understanding of the metabolism of the disaccharide. In addition, ADP-Glc PPase and GSase (mainly involved in glycogen synthesis) as well as UDP-Glc PPase were recombinantly produced with high purity after expression in *M. smegmatis* mc^2^155. This strategy was critical to solve a recalcitrant problem for the soluble expression of these enzymes in *E. coli*, which has been reported for many other mycobacterial proteins [53], [54], [65], [66].

**Fig. 5:**
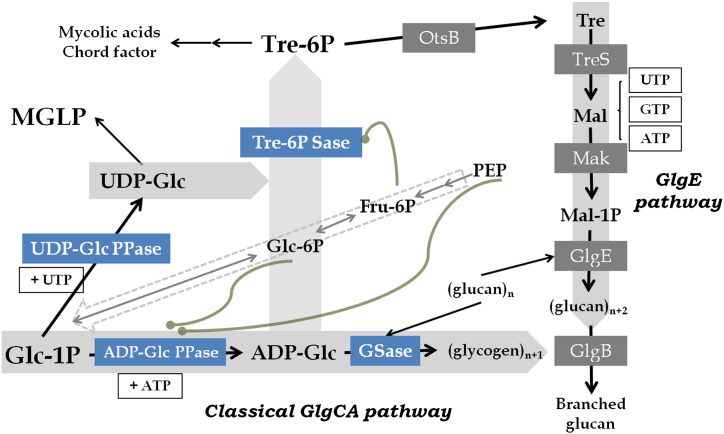
The metabolic pathways of *Mtb* that interconnect glycogen, Tre and other carbohydrates. The scheme includes links between pathways for glycogen, Tre (wide gray arrows) and MGLP. The enzymes characterized in this work (ADP-Glc PPase, UDP-Glc PPase, GSase and Tre-6P Sase) are shown in blue boxes. The green lines indicate the allosteric activation of ADP-Glc PPase and Tre-6P Sase. The wide-dashed arrow symbolizes the gluconeogenesis pathway and →→ indicates several enzymatic steps.

The kinetic and regulatory properties of the enzymes herein characterized are shown in Fig. 5 within the metabolic context of *Mtb*, where the pathways determining the fate of Glc-1P into oligo and polysaccharides, Tre, and glycogen are interlinked. The specificity determined for UDP-Glc PPase, ADP-Glc PPase and GSase support the occurrence of the classical ADP-Glc-dependent GlgCA pathway for glycogen synthesis, where ADP-Glc PPase is regulated by Glc-6P and PEP, two key metabolites of glycolysis. Thus, Glc-1P would be utilized to produce either UDP-Glc in a constant non-regulated manner or ADP-Glc in a regulated manner when levels of the glycolytic intermediates are increased. It is noteworthy that our results suggest that ADP-Glc would serve not only for glycogen synthesis but also to produce Tre-6P (Fig. 5). The importance of the sugar nucleotide for the accumulation of the polysaccharide agrees with previous works [13] demonstrating that a *glgC* knockout mutant of *Mtb* H37Rv accumulated 40–50% less glycogen and capsular glucan compared with the wild type strain. However, the promiscuity we determined for Tre-6P Sase to use ADP-Glc and to some extent UDP-Glc is a novel characteristic that necessitates a revised view of the essential OtsAB pathway in *Mtb* [67]. This new view also reinforces the critical metabolic node constituted by Glc-1P in the microorganism, as it is a key intermediate in the interconnection between Tre and glycogen metabolisms [28], [30], [32], [33], [64], as well as also being a key precursor for the synthesis of mycolic acids derivatives [68], [69]. Thus it seems likely that the constitutive production of UDP-Glc would serve the biosynthesis of MGLPs and Tre, which in turn feeds into glucan and cord factor biosynthesis. When the glycolytic intermediates PEP and Glc-6P build up, ADP-Glc production increases, which not only diverts flux into the GlgCA glycogen pathway but also increases flux into Tre production. The latter is also enhanced by the activation of Tre-6P Sase by Fru-6P.

The regulatory properties of the *Mtb* ADP-Glc PPase are distinct from those of other prokaryotes [11], but similar to the homologous protein from the related Gram-positive bacteria *M. smegmatis* [58] and *S. coelicolor* [27]. Despite some differences in the sensitivity to activation by Glc-6P and PEP, the fact that both ADP-Glc PPases from actinobacteria mainly respond to these effectors suggests the occurrence of similar domains involved in allosteric regulation given their close phylogenetic relatedness. The specificity toward effectors exhibited by the enzyme has commonalities with characteristics reported for the occurrence and modulation of other metabolic routes in mycobacteria. For example, *M. smegmatis* pyruvate kinase (catalyzing the conversion of PEP into pyruvate plus ATP) is activated by Glc-6P [70], and the hexose-P was reported as a key essential intermediate for mycobacterial metabolism [71].

It has been proposed that carbohydrates in *Mtb* may be utilized for anabolic rather than catabolic purposes during host infection [6]. This was based on observations that the organism: (i) lacks PEP carboxylase, which is functionally replaced by pyruvate carboxylase, and (ii) several key glycolytic enzymes (triose-P isomerase, phosphoglycerate kinase and glyceraldehyde-3-P dehydrogenase) are dispensable for growth on a source of carbohydrates [5], [72], [73]. In addition, recent studies on a (neo)glycolytic pathway found in *Mtb* have attracted much interest by shedding light on the importance that central metabolism has in the bacterium's biology, with new features (e.g. co-metabolism, re-routing or plasticity) being described [4], [6], [7], [8], [9], [74]. Thus, fatty acids seem to be actively catabolized to provide carbon and energy in mycobacteria, whereas carbohydrates are scavenged to provide biosynthetic precursors such as Glc-6P [3], [4], [6], [8], [72]. These metabolic peculiarities enhance the importance of Glc-6P and PEP [the metabolites located at the beginning/end of the (neo)glycolytic and gluconeogenic pathway (see Fig. 5)] as main allosteric activators of ADP-Glc PPase, since the classical GlgCA pathway for glycogen synthesis would be fully operative when levels of carbohydrates are being maintained high via anaplerosis.

It has been recently demonstrated that *Mtb* requires phosphorylated Glc to support mouse infection, suggesting the essentiality of the hexose for the bacterium's metabolism [74]. On the other hand, a mutant lacking phosphofructokinase activity showed that accumulation of Glc-6P and/or Fru-6P was detrimental for *Mtb* growing in anaerobic conditions [9], which is consistent with the toxicity of other sugar-P [64] or metabolic intermediates [75]. Taking into account these and our results, it could be speculated that there is another role for glycogen in *Mtb*. The polysaccharide could function as a carbon-buffer/capacitor, since an increment in the hexose-P pool could be directed to the polyglucan via the classical GlgCA pathway. It is noteworthy that in the close relative organism *Corynebacterium glutamicum*, glycogen was proposed as a carbon capacitor [76], [77].

Besides glycogen, extracellular α-glucan and methyl glucose polysaccharide (MGLP) are polymers playing critical roles for *Mtb* physiology [13]. Pathogenic mycobacteria are surrounded by a non-covalently bound capsule, whose major carbohydrate constituent is a glycogen like α-glucan. This cover plays a key role during the first stages of infection. Glycogen and α-glucan may even share in part a common biosynthetic route [14], [78], [79]. Additionally, glucosyl-3-phosphoglycerate synthase (GpgS) from *Mycobacterium bovis* BCG catalyzes glucosylglycerate synthesis by condensation of NDP-Glc and 3-phosphoglycerate [17], [80]. This molecule is the precursor for the biosynthesis of MGLP participating in modulation of fatty acids elongation [15], [16], [17]. GpgS utilizes both UDP-Glc and ADP-Glc, having a similar *V*_max_, although with a 6-fold higher affinity toward the former sugar nucleotide [17]. The MGLP molecule is predicted to be elongated by the glycosyltransferase Rv3032 which also utilizes NDP-Glc, but the substrate specificity has yet to be reported in detail. Thus, the glucosyl transferase activity required for the glucan backbone of these three macromolecules (glycogen, α-glucan and MGLP) [13], [15] would be originally supplied with glucose building blocks coming from ADP-Glc PPase and/or UDP-Glc PPase characterized in this work. UDP-Glc PPase affinity for Glc-1P is 10-fold higher than ADP-Glc PPase affinity for same substrate (in absence of allosteric effector). In this context, Glc-1P would be constantly used to synthesize UDP-Glc, while its fluctuating consumption by ADP-Glc PPase would be mainly modulated toward the enzyme allosteric regulation by levels of Glc-6P and PEP (Fig. 5).

*Mtb* has become a formidable pathogen by utilizing whatever carbon it can acquire to maximize its potential for growth. In order to achieve this, it must carefully regulate metabolic fluxes to key molecules involved in carbon storage (cytosolic glycogen), immune evasion (capsular alpha-glucan), the modulation of fatty acid biosynthesis (MGLP) and pathogenesis (Tre mycolates). Each of these molecules is originally generated via either ADP-Glc or UDP-Glc that are both generated from Glc-1P. We have shown how this critical node is controlled by the allosteric regulation of ADP-Glc PPase. More work is required to identify other regulatory nodes that no doubt exist.
