# Supplementary material for: Allosteric regulation of the partitioning of glucose-1-phosphate between glycogen and trehalose biosynthesis in Mycobacterium tuberculosis
Source: Biochim Biophys Acta Gen Subj. 2015 Jan;1850(1):13–21. doi: 10.1016/j.bbagen.2014.09.023 (PMC4331664; doi:10.1016/j.bbagen.2014.09.023)

**Supplemental Table I:** Oligonucleotides used in this work.

| Primer | Sequence | Restriction site |
| --- | --- | --- |
| Cfow | 5′-GGTACCATGAGAGAAGTCCCGCACGT-3′ | *Kpn*I |
| Crev | 5′‑CTGCAGGATCCAAACACCCTTGC-3′ | *Pst*I |
| Ufow | 5′‑GGATCCATGTCACGCCCAGAAGTAC-3′ | *Bam*HI |
| Urev | 5′‑CTGCAGCTGCTCTGTCAGACCCAGTG-3′ | *Pst*I |
| Afow | 5′‑GGTACCATGCGGGTGGCGATGTTGAC-3′ | *Kpn*I |
| Arev | 5′‑CTGCAGCGCGCACACCTTCCGGT-3′ | *Pst*I |
| Tfow | 5′‑ GGATCCATGGCTCCCTCGGGAGGCCA-3′ | *Bam*HI |
| Trev | 5′‑ AAGCTTGCCTTGGCCCCTCGGGTGTG-3′ | *Hind*III |

**Supplemental Figure 1: SDS-PAGE analysis of *Mtb* ADP-Glc PPase expression using the vector pET19b (*).** All expression assays were carried out at 23 ºC during 2 h with 0.1 mM IPTG. **(A)** Expression analysis in *E. coli* Rosetta. Lanes: 1, soluble fraction of cells transformed with [pET19b] without gene inserted (control); 2, molecular mass marker; 3, soluble fraction of cells transformed with [pET19b/*glgC*]; 4, insoluble fraction of cells transformed with [pET19b/*glgC*]. **(B)** ADP-Glc PPase expression using in *E. coli* Rosetta cells transformed with [pET19b/*glgC*] and grown in different culture media. Lanes: 1, molecular mass marker; 2, soluble fraction, growth in LB-Glc medium; 3, insoluble fraction, growth in LB-Glc medium; 4, soluble fraction, growth in TB medium; 5, insoluble fraction, growth in Terrific Broth medium; 6, soluble fraction, growth in M9 medium; 7, insoluble fraction, growth in M9 medium. **(C)** ADP-Glc PPase co-expressed with chaperones system pG‑KJE8 in *E. coli* BL21 (DE3) cells. Lanes: 1, molecular mass marker; 2, soluble fraction of cells transformed with [pET19b/*glgC*] plus [pG‑KJE8]; 3, insoluble fraction of cells transformed with [pET19b/*glgC*] plus [pG‑KJE8].


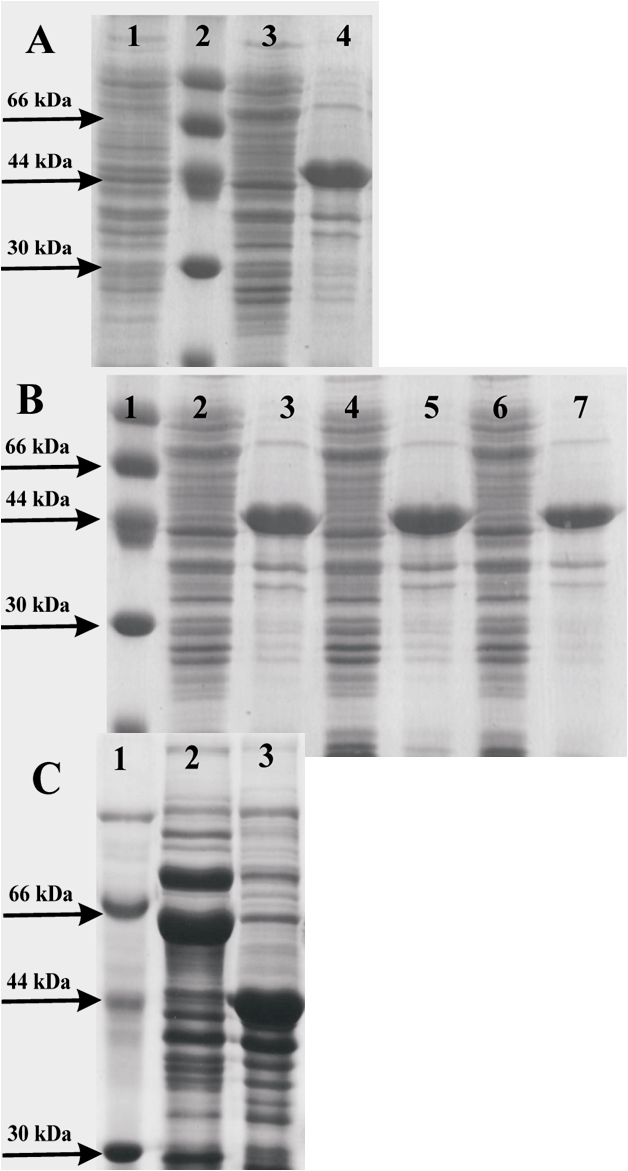


**(*)** Figures show results obtained with mycobacterial *glgC* gene cloned onto pET19 plasmid using *E. coli* Rosetta strain as a host (panel A) when different culture conditions were analyzed (panel B) or when co-expressed with chaperones (panel C), as stated under Materials and methods. Similar results were achieved with *glgC* when subcloned in the expression plasmids pET24; pET32 ; pRSET and pMAL and the *E. coli* strains Origami ; BL 21 (DE3) and Tunner (DE3) (not shown). In addition, the same behaviour was observed for mycobacterial *galU* and *glgA* expression assays in *E. coli* systems. In all cases, temperature and IPTG concentration ranged from 20 to 30 °C and 0.1 to 1 mM, respectively.

**Supplemental Figure 2: SDS-PAGE of purified recombinant enzymes.** From left to right: *Mtb* ADP-Glc PPase [expected mass 45 kDa including His-tag]* expressed in *M. smegmatis* mc^2^155, *Mtb* UDP-Glc PPase [33 kDa] expressed in *M. smegmatis* mc^2^155, *Mtb* GSase [43 kDa] expressed in *M. smegmatis* mc^2^155 and *Mtb* Tre-6P Sase [57 kDa] expressed in *E. coli.* The figure is a composite of individual gels stained with Coomassie‑Brilliant Blue and aligned according to the marker.


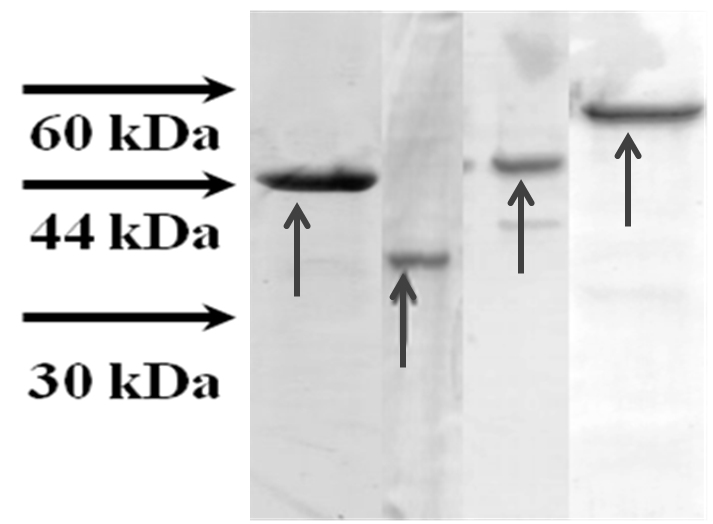

Supplement: Supplementary file 1 — Supplementary material. [file mmc1.docx]
